# HOXB13 and ALX4 induce SLUG expression for the promotion of EMT and cell invasion in ovarian cancer cells

**DOI:** 10.18632/oncotarget.3673

**Published:** 2015-04-10

**Authors:** Hong Yuan, Hiroaki Kajiyama, Satoko Ito, Dan Chen, Kiyosumi Shibata, Michinari Hamaguchi, Fumitaka Kikkawa, Takeshi Senga

**Affiliations:** ^1^ Department of Obstetrics and Gynecology, Nagoya University Graduate School of Medicine, Nagoya, 466-8550, Japan; ^2^ Division of Cancer Biology, Nagoya University Graduate School of Medicine, Nagoya, 466-8550, Japan

**Keywords:** EMT, HOXB13, ALX4, ovarian cancer, invasion

## Abstract

Homeoproteins, a family of transcription factors that have conserved homeobox domains, play critical roles in embryonic development in a wide range of species. Accumulating studies have revealed that homeoproteins are aberrantly expressed in multiple tumors and function as either tumor promoters or suppressors. In this study, we show that two homeoproteins, HOXB13 and ALX4, are associated with epithelial to mesenchymal transition (EMT) and invasion of ovarian cancer cells. HOXB13 and ALX4 formed a complex in cells, and exogenous expression of either protein promoted EMT and invasion. Conversely, depletion of either protein suppressed invasion and induced reversion of EMT. SLUG is a C2H2-type zinc-finger transcription factor that promotes EMT in various cell lines. Knockdown of HOXB13 or ALX4 suppressed SLUG expression, and exogenous expression of either protein promoted SLUG expression. Finally, we showed that SLUG expression was essential for the HOXB13- or ALX4-mediated EMT and invasion. Our results show that HOXB13/SLUG and ALX4/SLUG axes are novel pathways that promote EMT and invasion of ovarian cancer cells.

## INTRODUCTION

Deregulated expression or activation, as well as inactivation, of transcription factors is commonly observed in numerous types of cancer. Several pathways that are aberrantly activated in tumor cells eventually converge on the activation of sets of transcription factors to promote or repress target genes for tumor progression. For example, the AP-1 transcription factor, which is a dimeric complex comprising members of the JUN, FOS and ATF families, are frequently overexpressed in tumor cells, and their exogenous expression can promote tumor proliferation, invasion and metastasis [1]. In addition to AP-1 family proteins, other transcription factors, such as MYC, NF-κB and STAT family members, are associated with the malignant characteristics of numerous tumors [2]. Targeting these transcription factors using dominant-negative forms of proteins, siRNAs or small chemical inhibitors is a promising therapeutic strategy to combat cancer.

Homeoproteins are a family of transcription factors that have conserved homeobox domains. Homeoproteins bind to specific DNA elements via homeobox domains and regulate the expression of target proteins to control cell growth, differentiation and cell-cell interactions during embryonic development. Mutations in some homeobox genes in humans have been identified to induce malformations in body shape [3]. There are more than 200 proteins with homeobox domains in mammals, and they can be categorized into several subfamilies, such as DLX, PAX, MSX, and HOX [4]. Homeoproteins are highly conserved in various species, and their physiological functions are under intensive investigation, although it has been more than 30 years since the first discovery of the proteins.

Accumulating studies have revealed that the deregulated expression of homeoproteins is associated with the progression of various tumors [5–7]. Cellular transformation is associated with dedifferentiation so that homeoproteins that are expressed during embryonic development are re-expressed in some tumors. One of the well-known homeoproteins that is associated with the promotion or suppression of tumors is the HOX subfamily of proteins [8]. There are 39 HOX proteins in mammals, and they can be classified into four paralogous clusters (A, B, C and D), each of which is clustered on four different regions of chromosomes. Multiple HOX proteins are overexpressed in several cancers, and their expression induces proliferation, invasion, angiogenesis and chemoresistance [9]. In addition to HOX proteins, other homeoproteins, such as SATB1 [10, 11] and PITX2 [12, 13], promote tumor progression. Some homeoproteins have been reported to promote tumor invasion by inducing epithelial to mesenchymal transition (EMT) [14–18]. EMT is a series of events by which epithelial cells lose cell-cell adhesion and cell polarity, and acquire mesenchymal characteristics and morphology. SIX1 and LBX1 facilitate breast cancer metastasis by inducing EMT [19, 20]. Recent reports have indicated crucial functions of PRRX1 in promoting EMT for the progression of multiple cancers [21, 22]. These studies show the critical role of homeoproteins for the induction of EMT and tumor progression.

Ovarian cancer is a highly metastatic disease and the most lethal of the gynecologic malignancies. Previous studies have shown that homeoproteins, such as MSX2 and ALX1, induced EMT and promoted tumor invasion [23, 24]. In this report, we show that two homeoproteins, HOXB13 and ALX4, can induce the expression of the EMT-associated transcriptional factor SLUG and promote invasion and EMT of ovarian cancer cells.

## RESULTS

### Depletion of HOXB13 induces reversion of EMT and inhibits cell invasion

**Figure 1:**
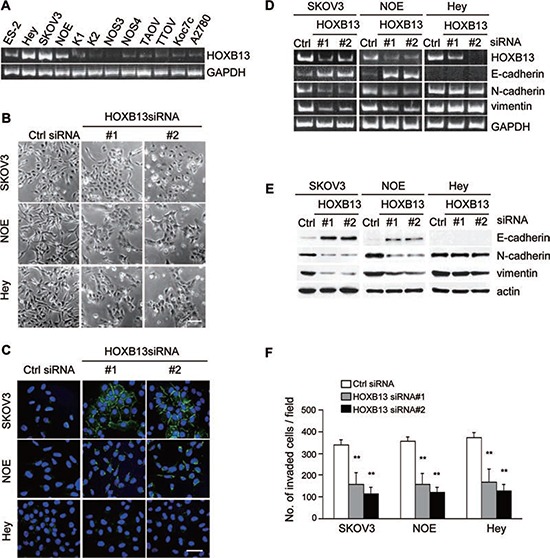
Depletion of HOXB13 induces reversion of EMT **A.** Expression of HOXB13 in ovarian cancer cell lines was examined using RT-PCR. **B.** Cells were transfected with siRNAs, and pictures were taken after 72 h to visualize the cellular morphology (Scale bar = 100 μm). **C.** Cells cultured on glass coverslips were transfected with siRNAs; 72 h later, cells were immunostained with anti-E-cadherin antibody and DAPI. Pictures were taken using a confocal fluorescence microscope (Green: E-cadherin, Blue: DAPI, Scale bar = 50 μm). **D.** Total RNA was extracted from siRNA-transfected cells, and the mRNA expression levels of the indicated genes were determined using RT-PCR. **E.** Following siRNA transfection, the expression of the indicated proteins was examined using immunoblotting. **F.** Cells were transfected with siRNA and then subjected to the *in vitro* invasion assay 72 h later. The graph indicates the average number of invaded cells per field. Three independent experiments were performed, and the data are shown as the mean ± SD (**P < 0.01).

When we performed a screen to search for genes that are associated with EMT and aggressive characteristics of ovarian cancer cells using a limited siRNA library [24], we noticed that the transfection of siRNAs targeting HOXB13 induced morphological changes of SKOV3 cells. A previous study reported that HOXB13 promoted ovarian cancer proliferation and conferred resistance to tamoxifen-mediated apoptosis [25], but the molecular mechanism of HOXB13-mediated tumor progression remains obscure. To gain further insight into the function of HOXB13 in ovarian cancer, we first examined the expression of HOXB13 mRNA in multiple ovarian cancer cell lines. Comparably high expression of HOXB13 was observed in HEY, SKOV3 and NOE (a new cell line of endometrioid carcinoma of ovary) cells (Fig. 1A and Fig. S1A); thus, we used these cells for further experiments. NOE cells were previously established from ovarian endometrioid carcinoma in our laboratory [26]. SKOV3, HEY and NOE cells cultured on glass coverslips were transfected with siRNAs; 72 h later, the cells were fixed, and cellular morphological changes and cell-cell contact was examined. Recovery of cell-cell adhesion was clearly observed in these cell lines by HOXB13 siRNA transfection (Fig. 1B). Immunostaining analysis revealed the accumulation of E-cadherin to the cell-cell contact site in SKOV3 cells, but not in HEY cells, in the absence of HOXB13 (Fig. 1C). Partial localization of E-cadherin at the cell-cell adhesion site was observed in HOXB13-depleted NOE cells (Fig. 1C).

We speculated that HOXB13 depletion induced mesenchymal to epithelial transition (MET), which is a reversion of EMT. To confirm the induction of MET, we examined the expression levels of epithelial (E-cadherin) and mesenchymal markers (vimentin and N-cadherin) using RT-PCR and immunoblot analysis. Consistent with the result obtained from immunofluorescence analysis, E-cadherin was up-regulated, and vimentin and N-cadherin were down-regulated by HOXB13 knockdown at the mRNA and protein levels in SKOV3 and NOE cells (Fig. 1D and 1E). However, there was no change in marker expression in HEY cells by HOXB13 knockdown (Fig. 1D and 1E). These results indicate that HOXB13 is indispensable to maintain the mesenchymal status of SKOV3 and NOE cells and that there are additional factors that maintain the mesenchymal phenotype in HEY cells other than HOXB13.

EMT is often associated with the invasive potential of cancer cells. We examined invasion of these cell lines in the absence of HOXB13 using Matrigel-coated Boyden chambers. Cells transfected with HOXB13 siRNAs showed significant reduction in cell invasion (Fig. 1F), indicating that HOXB13 is associated with the invasive potential of ovarian cancer cells.

### Depletion of ALX4 induces reversion of EMT and inhibits cell invasion

**Figure 2:**
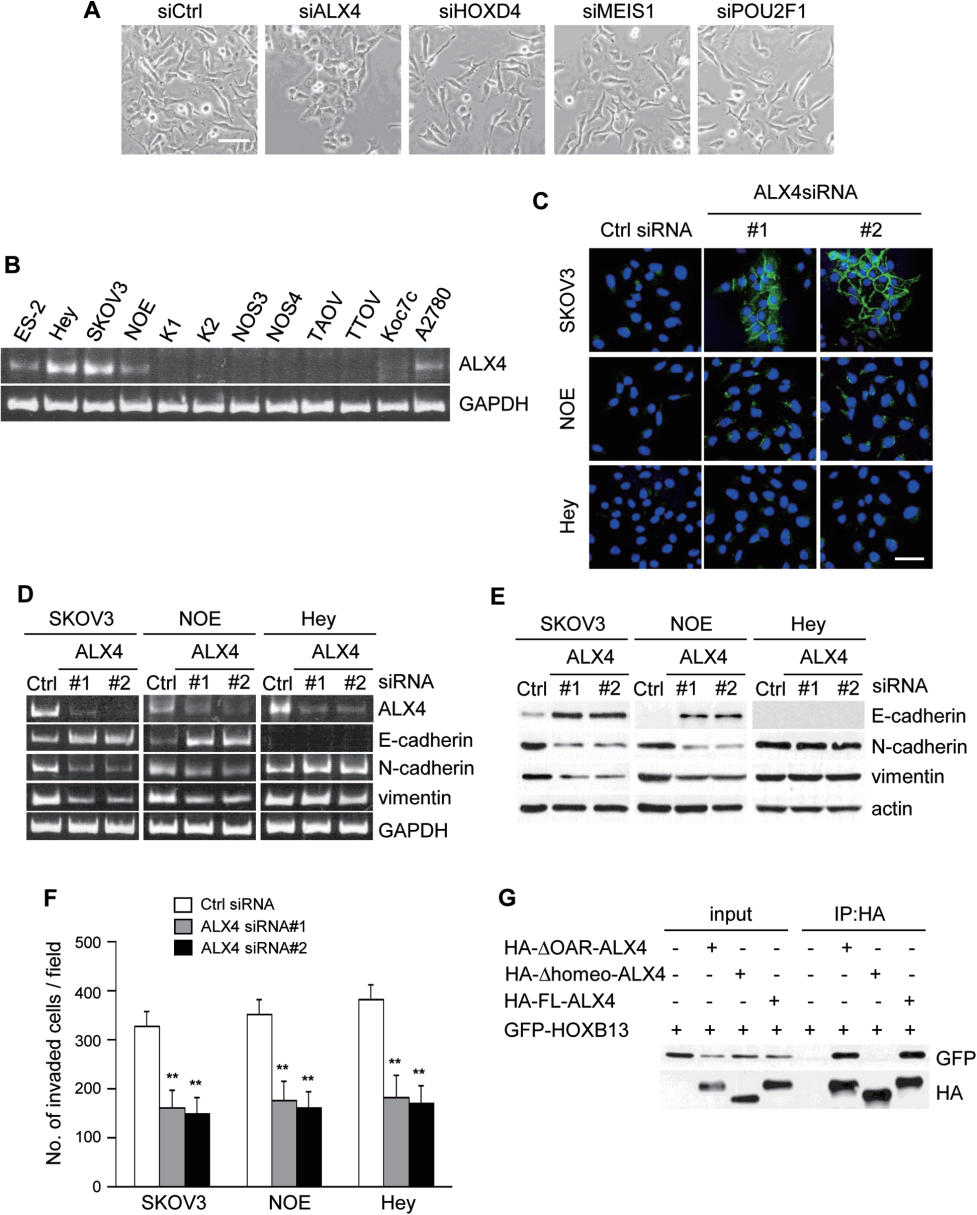
Depletion of ALX4 induces reversion of EMT **A.** Cells were transfected with siRNAs, and pictures were taken after 72 h to visualize the cellular morphology (Scale bar = 100 μm). **B.** Expression of ALX4 in ovarian cancer cell lines was examined using RT-PCR. **C.** Cells cultured on the glass coverslips were transfected with siRNAs; 72 h later, cells were immunostained with anti-E-cadherin antibody and DAPI. Pictures were taken using a confocal fluorescence microscope (Green: E-cadherin, Blue: DAPI, Scale bar = 50 μm). **D.** Total RNA was extracted from siRNA-transfected cells, and the mRNA expression levels of the indicated genes were determined using RT-PCR. **E.** Following siRNA transfection, the expression of the indicated proteins was examined using immunoblotting. **F.** Cells were transfected with siRNA and then subjected to the *in vitro* invasion assay 72 h later. The graph indicates the average number of invaded cells per field. Three independent experiments were performed, and the data are shown as the mean ± SD (**P < 0.01). **G.** The indicated combinations of proteins were transiently expressed in 293T cells and immunoprecipitated with anti-HA antibody. The immunoprecipitates were immunoblotted with anti-HA or anti-GFP antibody.

Homeoproteins often form homo- or heterodimers for the activation of target genes [27–30]. HOXB13 has been reported to interact with MEIS1 for the binding to specific DNA elements [31]. A previous large-scale analysis of protein-protein interactions using mammalian two-hybrid analyses revealed the possible interactions of HOXB13 with other homeoproteins, including ALX4, HOXD4 and POU2F1 [32, 33]. To explore whether these interacting partners play any role in the reversion of EMT, we suppressed the expression of ALX4, HOXD4, MEIS1 and POU2F1 in SKOV3 cells by siRNA transfection and examined the changes in cell morphology and expression of EMT markers. The mRNA level of each gene was significantly reduced by siRNA transfection (Fig. S2A). We found that the depletion of ALX4 induced morphological changes similar to those of HOXB13 depletion, although HOXD4, MEIS1 and POU2F1 knockdown did not show any morphological changes indicative of MET (Fig. 2A).

We examined level of ALX4 mRNA in ovarian cancer cell lines. ALX4 was expressed in HEY, NOE and SKOV3 cells (Fig. 2B and Fig. S1B); thus, we depleted ALX4 in SKOV3, HEY and NOE cells and examined E-cadherin localization. Similar to HOXB13-depleted cells, clear accumulation of E-cadherin to the cell-cell contact sites was observed only in SKOV3 cells by ALX4 knockdown (Fig. 2C). However, ALX4 depletion in HEY cells and NOE cells induced the recovery of cell-cell adhesion, and the cellular morphology became similar to that of epithelial cells (Fig. S2B). We investigated the expression of markers for EMT using RT-PCR and immunoblot analysis. The up-regulation of E-cadherin, as well as the down-regulation of N-cadherin and vimentin, was observed in SKOV3 and NOE cells but not in HEY cells by ALX4 knockdown (Fig. 2D and 2E). The expression of marker proteins in SKOV3 cells was not affected by the depletion of HOXD4, MEIS1 and POU2F1 (Fig. S2C). We next investigated the invasive potential of SKOV3, NOE and HEY cells in the absence of ALX4. As shown in Fig. 2F, invasion of these cells was significantly suppressed by ALX4 depletion.

Finally, we tested whether ALX4 and HOXB13 can interact in cells. ALX4 has a homeodomain in the central region and OAR domain at the C-terminal end. We created ALX4 that is deleted of the homeodomain (Δhomeo-ALX4; deleted of aa 215–273) or OAR domain (ΔOAR-ALX4; deleted of aa 387–406) and determined the region critical for the interaction. HA-tagged full length (FL) ALX4 and a deletion mutant of ALX4 were expressed in 293T cells together with GFP-HOXB13, and then the cells were immunoprecipitated with anti-HA-antibody. HOXB13 was co-precipitated with FL- and ΔOAR-ALX4 but not with Δhomeo-ALX4 (Fig. 2G), indicating that HOXB13 and ALX4 can form a heterodimer through the homeobox domain.

### Exogenous expression of HOXB13 and ALX4 induces EMT and promotes invasion

**Figure 3:**
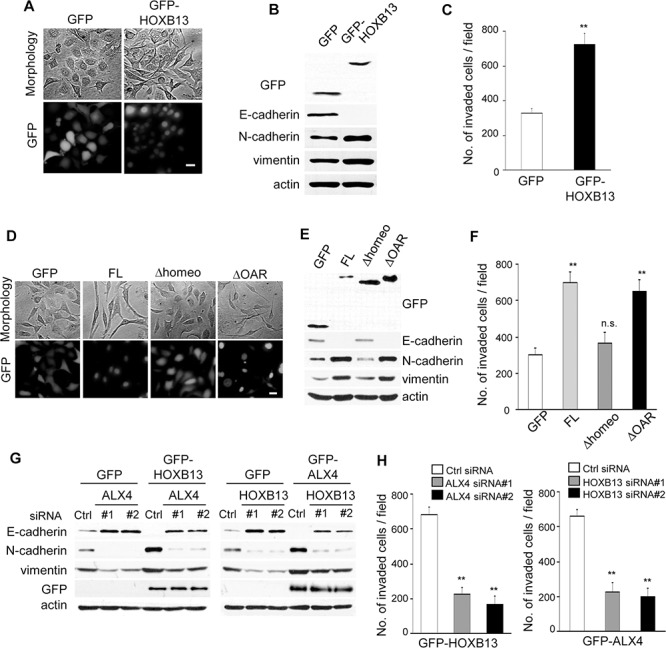
Exogenous expression of HOXB13 and ALX4 promote EMT **A.** SKOV3 cells that constitutively expressed GFP or GFP-HOXB13 were established by retrovirus infection (Scale bar = 20 μm). **B.** The expression of the indicated proteins was examined using immunoblotting. **C.** The graph indicates the average number of invaded cells per field. Three independent experiments were performed, and the data are shown as the mean ± SD (**P < 0.01). **D.** SKOV3 cells that constitutively expressed the indicated proteins were generated by retrovirus infection (Scale bar = 20 μm). **E.** The expression of the indicated proteins was examined using immunoblotting. **F.** The graph indicates the average number of invaded cells per field. Three independent experiments were performed, and the data are shown as the mean ± SD (**P < 0.01). **G.** GFP, GFP-HOXB13 and GFP-ALX4 SKOV3 cells were transfected with the indicated siRNAs; 72 h later, the expression of the indicated proteins was examined using immunoblotting. **H.** The graph indicates the average number of invaded cells per field. Three independent experiments were performed, and the data are shown as the mean ± SD (**P < 0.01).

We next examined the effect of the exogenous expression of HOXB13 in SKOV3 cells. Cells that constitutively expressed GFP or GFP-tagged full-length HOXB13 were generated by retrovirus infection. As shown in Fig. 3A, cell-cell adhesion was disrupted, and cells became more elongated by HOXB13 expression. Consistently, immunoblot analysis showed the down-regulation of E-cadherin as well as the up-regulation of N-cadherin and vimentin expression (Fig. 3B). Cell invasion was promoted by HOXB13 expression as well (Fig. 3C).

We tested whether ALX4 expression had a similar effect on SKOV3 cells. GFP-tagged full-length ALX4, as well as the deletion mutant of ALX4, was expressed in SKOV3 cells, and changes in cell morphology and marker protein expression were examined. Disruption of cell-cell adhesion was observed in FL-ALX4 and ΔOAR-ALX4 cells, whereas no clear morphological changes were observed by GFP and Δhomeo-ALX4 expression (Fig. 3D). Immunoblot analysis confirmed the induction of EMT by either FL-AXL4 or ΔOAR-ALX4 expression (Fig. 3E). Consistently, both FL-AXL4 and ΔOAR-ALX4 SKOV3 cells showed enhanced invasion compared with GFP and Δhomeo-ALX4 SKOV3 cells (Fig. 3F). Promotion of EMT by either HOXB13 or ALX4 expression was also observed in another ovarian cancer cell line, NOS3 (Fig. S3).

We next examined whether ALX4 expression was required for the promotion of EMT and cell invasion by HOXB13 expression. GFP-HOXB13 SKOV3 cells were transfected with ALX4 siRNAs; 72 h later, the expression of marker proteins was examined using immunoblotting. Depletion of ALX4 clearly restored the expression of E-cadherin in GFP-HOXB13 SKOV3 cells (Fig. 3G). In addition, vimentin and N-cadherin expression in GFP-HOXB13 SKOV3 cells was suppressed by ALX4 knockdown (Fig. 3G). HOXB13 knockdown in GFP-ALX4 SKOV3 cells similarly abolished the ALX4-induced promotion of EMT (Fig. 3G). Enhanced cell invasion by GFP-ALX4 and GFP-HOXB13 was also diminished by the depletion of HOXB13 and ALX4, respectively (Fig. 3H). These results indicate that ALX4-mediated EMT and invasion of SKOV3 and NOE cells are dependent on the existence of HOXB13 and vice versa.

### ALX4 and HOXB13 promote the expression of SLUG

**Figure 4:**
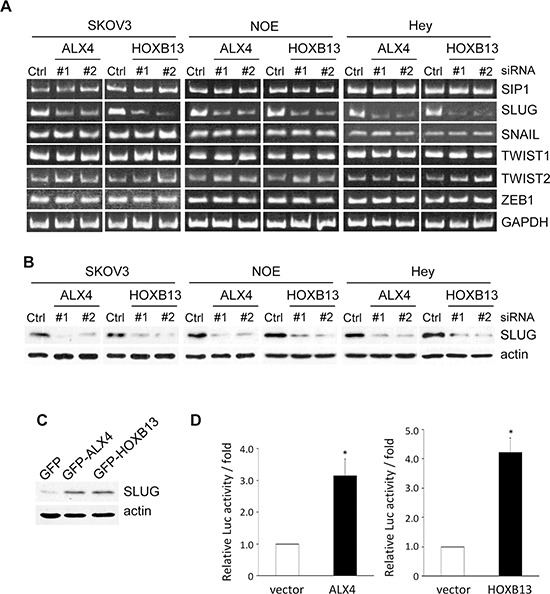
ALX4 and HOXB13 induce SLUG expression **A.** Cells were transfected with siRNAs; 72 h later, the mRNA levels of the indicated transcription factors were examined using RT-PCR. **B.** Expression of SLUG in siRNA-transfected cells was examined using immunoblotting. **C.** The expression of SLUG in GFP-HOXB13 and GFP-ALX4 SKOV3 cells was examined using immunoblotting. **D.** 293T cells were co-transfected with the HOXB13 or ALX4 expression vector together with the pGL4-SLUG/promoter and pRK-Luc expression vectors. Three independent experiments were performed, and the relative luciferase activity is indicated (**P < 0.01).

To examine the molecular mechanisms of ALX4- and HOXB13-mediated EMT, we searched for transcriptional factors whose expression levels were regulated by either protein. SKOV3 cells were transfected with siRNAs; 72 h later, mRNA was extracted, and the levels of EMT-related transcriptional factors were evaluated using RT-PCR. Among the transcriptional factors we examined, the level of SLUG mRNA was significantly reduced in SKOV3 cells transfected with either ALX4 or HOXB13 siRNA (Fig. 4A). The expression of SLUG protein was similarly suppressed in the absence of ALX4 or HOXB13 (Fig. 4B). Conversely, the exogenous expression of either protein promoted the expression of SLUG (Fig. 4C). We performed luciferase assays to determine whether SLUG promoter activity was regulated by the expression of ALX4 and HOXB13. 293T cells were transiently co-transfected with a reporter construct in which the human SLUG promoter region was cloned upstream of firefly luciferase (pGL4-SLUG/promoter) together with a plasmid encoding either GFP-ALX4 or GFP-HOXB13. The exogenous expression of either protein increased SLUG promoter activity approximately 3~4-fold (Fig. 4D). These results indicate that both ALX4 and HOXB13 can induce the transcription of the SLUG gene.

### SLUG expression is essential for ALX4- and HOXB13-mediated EMT and invasion

**Figure 5:**
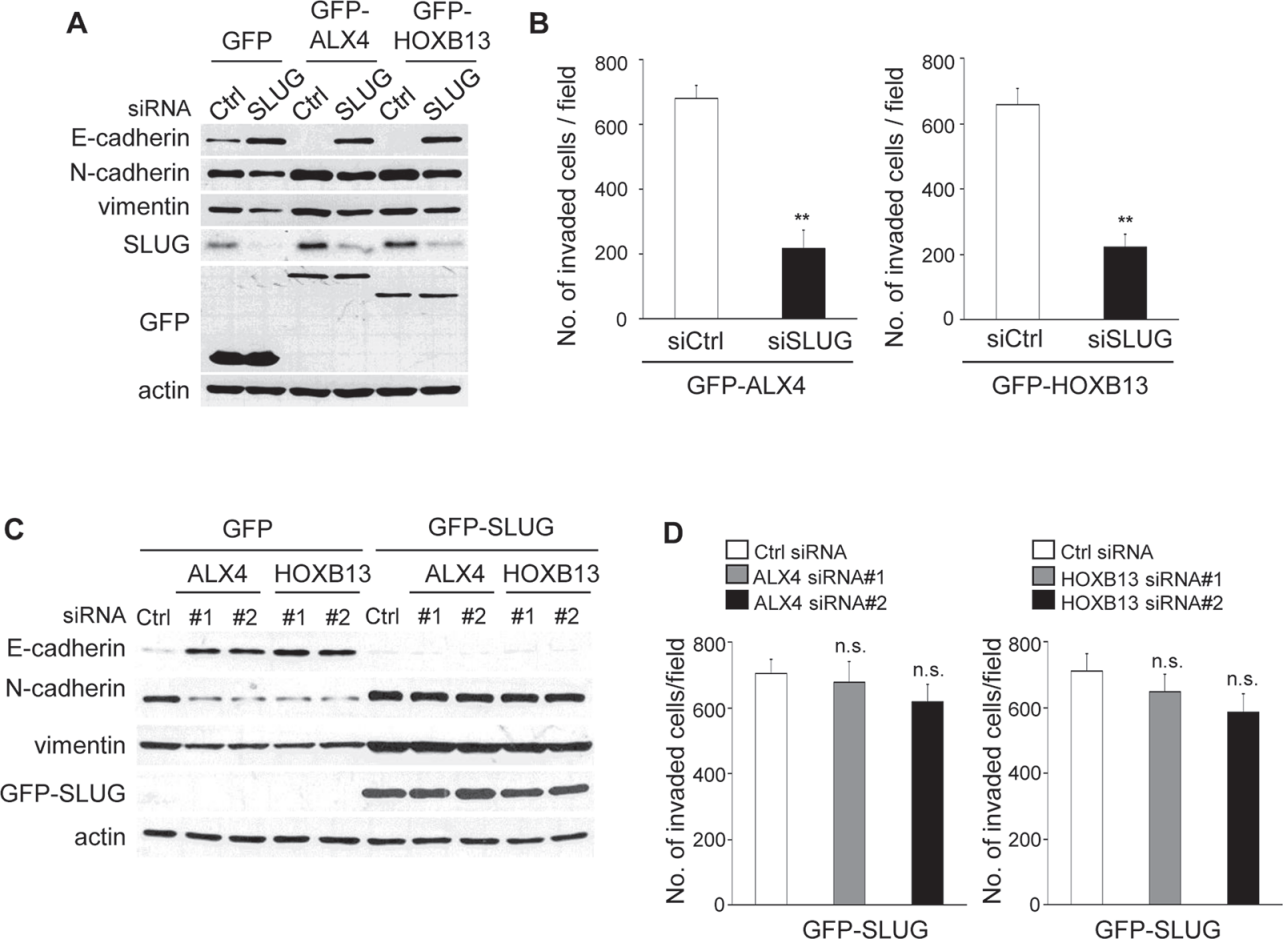
SLUG expression is critical for ALX4- and HOXB13-mediated EMT and cell invasion **A.** GFP-HOXB13 or GFP-ALX4 SKOV3 cells were transfected with SLUG siRNA; 72 h later, the expression of the indicated proteins was determined using immunoblotting. **B.** The graph indicates the average number of invaded cells per field. Three independent experiments were performed, and the data are shown as the mean ± SD (**P < 0.01). **C.** GFP-SLUG SKOV3 cells were transfected with siRNAs; 72 h later, the expression of the indicated proteins was determined using immunoblotting. **D.** The graph indicates the average number of invaded cells per field. Three independent experiments were performed, and the data are shown as the mean ± SD (n.s., not significant).

To determine whether SLUG expression is critical for ALX4- and HOXB13-induced EMT and cell invasion, we depleted SLUG expression in GFP-ALX4 and GFP-HOXB13 SKOV3 cells. Expression of E-cadherin in GFP-ALX4 or GFP-HOXB13 SKOV3 cells was clearly restored by SLUG siRNA transfection (Fig. 5A). Enhanced cell invasion by the exogenous expression of either protein was suppressed by SLUG knockdown as well (Fig. 5B).

To further confirm the crucial function of SLUG, we established SKOV3 cells that constitutively expressed GFP-SLUG. If SLUG expression is critical for the ALX4- or HOXB13-induced EMT, knockdown of either protein in SLUG-expressing SKOV3 cells would not induce reversion of EMT. GFP-SLUG SKOV3 cells were transfected with ALX4 or HOXB13 siRNA, and the expression of marker proteins was determined via immunoblot analysis. As shown in Fig. 5C, knockdown of ALX4 or HOXB13 did not affect the expression of E-cadherin, N-cadherin, vimentin in GFP-SLUG SKOV3 cells. In addition, invasion of GFP-SLUG SKOV3 cells was not suppressed by depletion of either protein (Fig. 5D). These results show that SLUG expression is critical for ALX4- and HOXB13-mediated EMT and cell invasion.

## DISCUSSION

Recent studies have revealed that HOXB13 is associated with the progression or suppression of multiple cancers. Extensive analysis has revealed that mutations in the HOXB13 gene are associated with a significantly increased risk of hereditary prostate cancer. The most commonly observed mutation is the substitution of glycine at amino acid residue 84 to glutamic acid (G84E), but the mechanism by which the mutation confers risk has not yet been determined [34, 35]. HOXB13 promotes or represses prostate cancer cell proliferation depending on the cellular context, such as with androgen receptor expression [36]. HOXB13 has opposing functions in other cancers as well. HOXB13 has a tumor-suppressive function in colorectal cancer, renal cell carcinoma and malignant melanoma [37–39]. By contrast, HOXB13 is overexpressed in tumors, such as breast, cervical and ovarian cancers, as well as hepatocellular carcinoma [40–43]. In ovarian cancer cells, a previous study showed that HOXB13 promoted cancer cell proliferation *in vivo* and conferred resistance to tamoxifen-mediated apoptosis [25]. In this report, we showed that the depletion of HOXB13 induced reversion of EMT and suppressed invasion of ovarian cancer cells. In addition, exogenous expression of HOXB13 promoted EMT and invasion of SKOV3 cells. These results indicate a possible role of HOXB13 for the promotion of EMT and invasion in ovarian cancer. HOXB13 is overexpressed in other cancers; thus, HOXB13 may induce EMT in multiple cancers for the promotion of invasion and metastasis.

ALX4 is expressed in the mesenchymal cells of bones, hair, teeth, limbs, whiskers and mammary gland during development [44–46]. Targeted deletion of ALX4 resulted in mice with multiple abnormalities, such as polydactyly, a defective craniofacial structure and body wall closure defects [44, 47]. Loss of ALX4 function in humans is associated with defects in craniofacial development [48, 49]. In addition to the critical function of ALX4 in development, recent studies have reported the correlation of ALX4 expression with cancer. Hypermethylation of the ALX4 gene was associated with tumorigenesis and prognosis in colorectal cancer [50]. In lung cancer, ALX4 expression was silenced by hypermethylation, and ectopic expression of ALX4 suppressed proliferation of lung cancer cells *in vitro* and *in vivo* [51]. By contrast, ALX4 was strongly expressed in a subtype of medulloblastoma, which is the most common pediatric brain tumor [52]. We showed that ALX4 had tumor-promoting function by promoting EMT and invasion in ovarian cancer cells. These results suggest that ALX4 has a tumor-promoting or tumor-suppressive function depending on the type of cancer.

Homeoproteins are known to homo- or hetero-dimerize with other homeoproteins to bind to specific DNA elements for the transcription of target genes. For example, homeoproteins such as HOXA13 and PAX3 form a homodimer for the transcription of target proteins [28, 29]. Several HOX proteins are known to bind PBX or MEIS homeoproteins for the stabilization of the DNA-protein complex as well as for transcriptional activation [27, 31]. Previous studies using the mammalian two-hybrid technique identified numerous interactions between homeoproteins [33]; thus, there may be a huge variety of heterodimers of homeoproteins for the regulation of the complicated processes of development. Our immunoprecipitation analysis demonstrated that ALX4 interacts with HOXB13 via the homeobox domain in cells. Either HOXB13 or ALX4 can also form a homodimer in cells (Fig. S4); therefore, we are not certain whether HOXB13 and ALX4 function as homodimer or heterodimer in cells. They may form a heterodimer to induce SLUG expression and EMT or either protein of a homodimer may synergistically activate different pathways for the promotion of EMT and invasion.

EMT is induced by various signal pathways initiated by extracellular stimuli or the activation of oncogenes, but these signals eventually promote the expression of some critical transcription factors to suppress E-cadherin expression associated with cellular morphological changes [53]. SLUG is one of the critical regulators for EMT, and its expression alone can confer the mesenchymal phenotype in many epithelial cells. SLUG is overexpressed in multiple cancers and is associated with the malignant characteristics of tumor cells [54, 55]. We showed that both HOXB13 and ALX4 promoted the expression of SLUG. Depletion of SLUG abolished HOXB13- and ALX4-mediated EMT, and SLUG-expressing cells were resistant to the reversion of EMT by either HOXB13 or ALX4 depletion. These results clearly show that EMT and cell invasion induced by either HOXB13 or ALX4 is dependent on the expression of SLUG. Homeoproteins have been reported to regulate the expression of EMT-related transcription factors. LBX1 and ALX1, a paralog of ALX4, induced SNAIL expression [19, 24], and DLX4 up-regulated TWIST for EMT induction [57]. Interconnections between homeoproteins and EMT-related transcription factors may play diverse roles in development and tumor progression.

In summary, we have shown that two homeoproteins, HOXB13 and ALX4, are associated with EMT and invasion of ovarian cancer cells. In addition, HOXB13- and ALX4-mediated EMT, as well as invasion, are dependent on the expression of SLUG. A complicated network of transcription factors plays crucial roles in tumor progression. Further analysis of the homeobox proteins, as well as EMT-related transcription factors, may reveal the novel molecular basis for the promotion of ovarian cancer.

## MATERIALS AND METHODS

### Cells, antibodies and chemicals

All of the ovarian cancer cells were cultured in RPMI supplemented with 10% FBS and antibiotics. K1, K2, NOE, NOS3, NOS4, TTOV and TAOV cells were established at the Department of Obstetrics and Gynecology, Nagoya University Graduate School of Medicine [26]. 293T cells were maintained in DMEM supplemented with 10% FBS with antibiotics. Antibodies were obtained from the following companies: Anti-E-cadherin, anti-N-cadherin, anti-vimentin antibodies: BD Biosciences, San Jose, CA, USA; anti-β-actin, anti-vinculin antibodies: Sigma-Aldrich, St. Louis, MO, USA; anti-GFP antibody: Neuro Mab, Davis, CA, USA; anti-Snail antibodies: Cell Signaling, Danvers, MA, USA. Rhodamine-conjugated phalloidin was obtained from Invitrogen and DAPI from Dojindo (Kumamoto, Japan).

### Plasmids

Full-length ALX4, HOXB13 and SLUG were amplified from a cDNA library derived from SKOV3 cells and cloned into the pQCXIP retrovirus vector (Clontech, Mountain View, CA, USA) with an N-terminal GFP tag. Deletion constructs for ALX4 were generated using PCR.

### RT-PCR

Cells were transfected with siRNA; 72 h later, total RNA was extracted using the RNeasy Mini Kit (Qiagen, Venlo, Netherlands). cDNA was generated using PrimeScript Reverse Transcript (TAKARA, Tokyo, Japan), and PCR was performed using specific primers for each gene.

### siRNA transfection

The sequences of the siRNAs used to suppress ALX4, HOXB13 and SLUG expression were 5′-GCUGAGACUUGCGUCUCUUTT-3′ (ALX4#1), 5′-GCUCUUCUCCACACAGCUUTT-3′ (ALX1#2), 5-GCACUUUAGAAACCGCUUUTT-3′ (HOXB13#1), 5-CCUUGCAUACUUAGCCCUUTT-3′ (HOXB13#2) and 5′-CGCGAACUCAGGUGCCUUAAA-3′ (SLUG). The sequences of siRNAs for other homeobox genes are 5′-CCCUUACCCUUCUGAAGAATT-3′ (MEIS1), 5′-GCCAGCAUUUACAGCCGAUTT-3′ (HOXD4), and 5′-CCUUCAAACAAAGACGAAUTT-3′ (POU2F1). The control siRNA sequence that targeted luciferase was 5′-CUUACGCUGAGUACUUCGATT-3′. siRNAs were obtained from Sigma. Cells were transfected with 20 nM of siRNA using Lipofectamine RNAiMAX (Invitrogen, Carlsbad, CA, USA) according to the manufacturer's instructions.

### Generation of stable cell lines

293T cells were transfected with the pQCXIP vector encoding each gene, as well as the pVPack-GP and pVPack-Ampho vectors (Stratagene, Tokyo, Japan). The culture supernatant was collected 48 h later and applied to SKOV3 cells with 2 μg/ml of polybrene (Sigma-Aldrich). Cells were cultured for 24 h, and then 1 μg/ml of puromycin (Sigma-Aldrich) was added to select for infected cells.

### Immunofluorescence analysis

Cells were grown on glass coverslips, fixed with ice-cold methanol:acetone (1:1) for 10 min, and blocked with phosphate-buffered saline (PBS) containing 7% fetal bovine serum for 30 min. Cells were incubated with primary antibody in PBS for 1 h, washed three times with PBS, incubated with Alexa Fluor 488 or Alexa Fluor 594-labeled secondary antibody (Invitrogen) in PBS for 1 h, and then analyzed using a confocal fluorescence microscope (FV1000-D, Olympus, Tokyo, Japan).

### Immunoblot analysis

Cells were lysed with Laemmli sample buffer (20% glycerol, 135 mM Tris-HCl pH 6.8, 4% SDS, 10% 2-mercaptoethanol, 0.003% BPB) and boiled for 5 minutes. The protein concentration of each lysate was measured using the RC-DC Protein Assay (Bio-Rad Laboratories, Hercules, CA, USA). Equal quantities of protein from each cell lysate were separated on SDS-polyacrylamide electrophoresis (SDS-PAGE) gels and transferred to PVDF membranes (Millipore, Billerica, MA, USA). The membranes were blocked with 1% skim milk, incubated with each primary antibody for 1 h, washed with TBS-T buffer (10 mM Tris-HCl pH 7.4, 150 mM NaCl, 0.05% Tween 20) and incubated with secondary antibodies. The proteins were visualized using enhanced chemiluminescence (GE Healthcare BioSciences, Uppsala, Sweden).

### Invasion assay

To measure cell invasion using Boyden chambers, the filter was pre-coated with Matrigel, and 1 × 10^5^ cells were seeded onto the upper surface of the chamber. Fourteen hours after seeding, cells were fixed with 70% methanol and stained with 0.5% crystal violet. Cells that invaded the lower surface of the filters were counted in five randomly selected fields. Three independent experiments were performed.

### Luciferase assay

The previously reported promoter region for SLUG (−516/+116) was amplified and cloned into the pGL4 vector (Promega, Madison, WI, USA). 293T cells were transfected with the pGL4-Snail/promoter together with pQCXIP-GFP-ALX4 or pQCXIP-GFP-ALXHOXB13 and pRTK-Luc to normalize the transfection efficiency. Forty-eight hours later, the activities of Firefly luciferase and Renilla luciferase were measured using the Dual Luciferase Reporter Assay System (Promega). Luciferase activity was measured in triplicate, and three independent experiments were performed.

## SUPPLEMENTARY FIGURES


