# Supplementary material for: HOXB13 and ALX4 induce SLUG expression for the promotion of EMT and cell invasion in ovarian cancer cells
Source: Oncotarget. 2015 Apr 10;6(15):13359–70. doi: 10.18632/oncotarget.3673 (PMC4537020; doi:10.18632/oncotarget.3673)
Supplement: Supplementary file 1 [file oncotarget-06-13359-s001.pdf]

SUPPLEMENTARY FIGURES

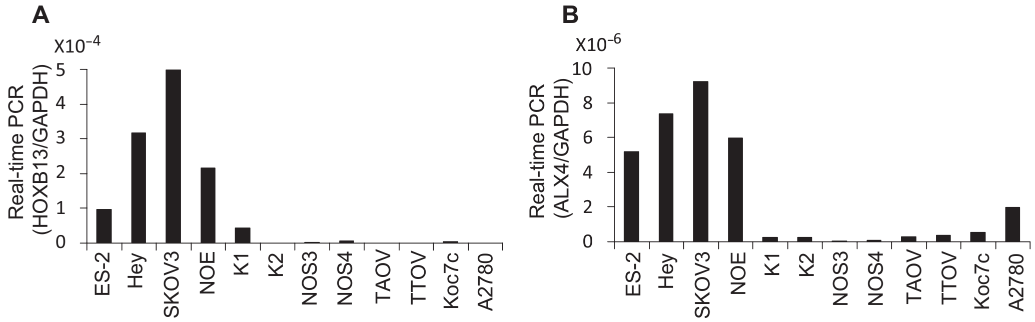

Supplementary Figure S1: A. The relative expression of HOXB13 mRNA normalized to GAPDH mRNA in ovarian cancer cell lines was examined by real time PCR analysis. B. The relative expression of ALX4 mRNA normalized to GAPDH mRNA in ovarian cancer cell lines was examined by real time PCR analysis.

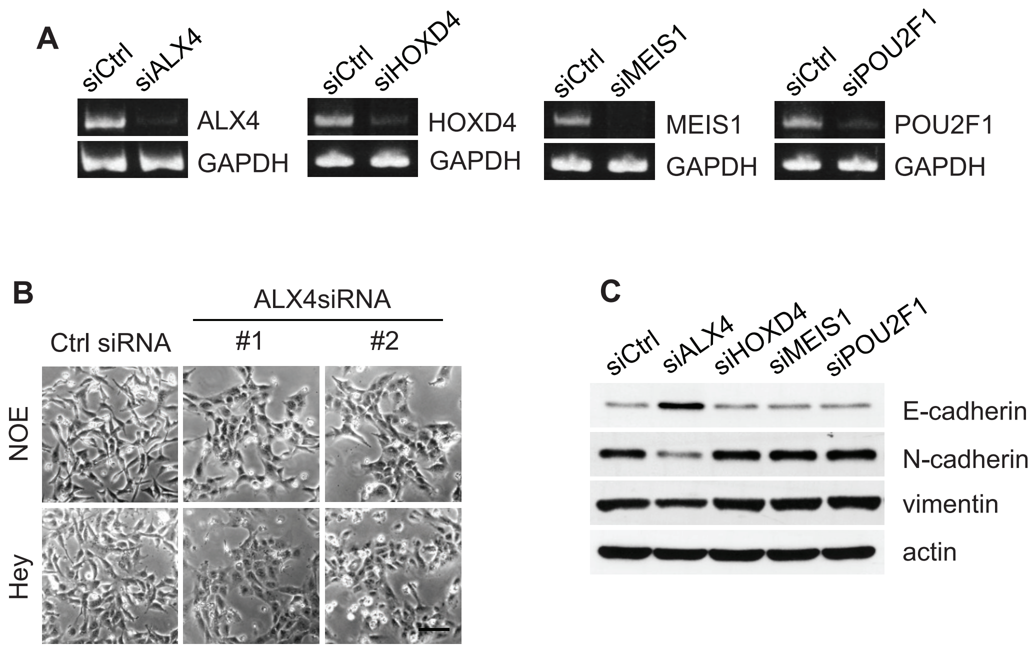

Supplementary Figure S2: A. Total RNA was extracted from siRNA-transfected SKOV3 cells, and the mRNA expression levels of the indicated genes were determined using RT-PCR. B. Cells were transfected with siRNAs, and pictures were taken after 72 h to visualize the cellular morphology. **C.** SKOV3 cells were transfected with siRNAs; 72 h later, the expression of the indicated proteins was examined using immunoblotting.

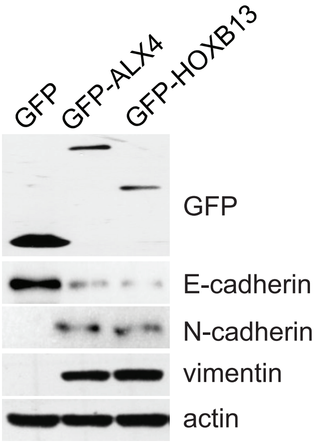

Supplementary Figure S3: NOS3 cells that constitutively expressed GFP, GFP-ALX4 and GFP-HOXB13 were established by retrovirus infection. Expression of indicated marker proteins was examined by immunoblot.

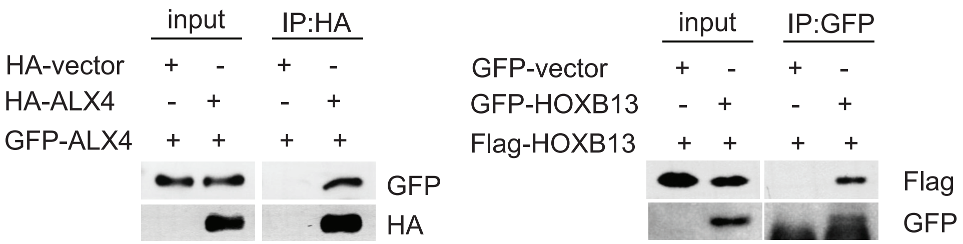

Supplementary Figure S4: 293T cells were transfected with the indicated combinations of plasmids; 24 h later, the cells were lysed and immunoprecipitated with anti-HA or anti-GFP antibody. The immunoprecipitates were blotted with the indicated antibodies.
